# Supplementary material for: Admission screening for extended-spectrum cephalosporin-resistant and carbapenem-resistant Enterobacterales colonization at a referral hospital in Botswana: A one-year period-prevalence survey, 2022–2023
Source: PLOS Glob Public Health. 2025 Oct 28;5(10):e0005018. doi: 10.1371/journal.pgph.0005018 (PMC12561989; doi:10.1371/journal.pgph.0005018)
Supplement: S1 Table — (DOCX) [file pgph.0005018.s001.docx]

Supplementary Table 1: Association of participant demographics and exposure with ESCrE/CRE *Enterobacter* spp. colonization among patients admitted to a tertiary care hospital, Botswana, 2022-2023.

| **Variable** | **Category** | **Observations n= 802 (%)** | ***Enterobacter* spp. n=13 (%)** | **Non-*Enterobacter* n=789 (%)** | **Odds Ratio (95% CI)** | **P-value**** |
| --- | --- | --- | --- | --- | --- | --- |
| Sex | Female | 382 (47.6) | 5 (1.3) | 377 (98.7) | REF | 0.50 |
|  | Male | 420 (52.4) | 8 (1.9) | 412 (98.1) | 1.46 (0.47-4.51) |  |
| Age (years) | 18-50 | 270 (33.7) | 4 (1.5) | 266 (98.5) | REF | 0.78 |
|  | <1 | 158 (19.7) | 3 (1.9) | 155 (98.1) | 1.29 (0.28-5.83) |  |
|  | 1-5 | 196 (24.4) | 3 (1.5) | 193 (98.5) | 1.03 (0.23-4.67) |  |
|  | 6-17 | 82 (10.2) | 0 (0.0) | 82 (100.0) | -- |  |
|  | >50 | 94 (11.7) | 3 (3.2) | 91 (96.8) | 2.19 (0.48-9.98) |  |
| Pre-existing chronic disease* | Not known | 769 (95.9) | 12 (1.6) | 757 (98.4) | REF | 0.56 |
|  | Known | 33 (4.1) | 1 (3.0) | 32 (97.0) | 1.97 (0.25-15.63) |  |
| Indwelling device | No | 764 (95.3) | 11 (1.4) | 753 (98.6) | REF | 0.14 |
|  | Yes | 38 (4.7) | 2 (5.3) | 36 (94.7) | 3.80 (0.81-17.80) |  |
| Antibiotics  (past 30 days) | No | 648 (80.8) | 10 (1.5) | 638 (98.5) | REF | 0.68 |
|  | Yes | 147 (18.33) | 3 (2.0) | 144 (98.0) | 1.33 (0.36-4.89) |  |
|  | Not sure | 7 (0.87) | 0 (0.0) | 7 (100.0) | -- |  |
| Cephalosporin (past 30 days) | No | 740 (92.3) | 11 (1.5) | 729 (98.5) | REF | 0.35 |
|  | Yes | 62 (7.7) | 2 (3.2) | 60 (96.8) | 2.21 (0.48-10.20) |  |
| Carbapenem  (past 30 days) | No | 800 (99.8) | 13 (1.6) | 787 (98.4) | REF | -- |
|  | Yes | 2 (0.2) | 0 (0.0) | 2 (100.0) | -- |  |
| Antiretroviral drugs (past 30 days) | No | 761 (94.9) | 12 (1.6) | 749 (98.4) | REF | 0.69 |
|  | Yes | 41 (5.1) | 1 (2.4) | 40 (97.6) | 1.56 (0.20-12.30) |  |
| Hospitalized in past 6 months | No | 693 (86.4) | 9 (1.3) | 684 (98.7) | REF | 0.26 |
|  | Yes | 103 (12.8) | 3 (2.9) | 100 (97.1) | 2.28 (0.61-8.56) |  |
| Referred from a clinic or hospital | No | 98 (12.2) | 1 (1.0) | 97 (99.0) | REF | 0.59 |
|  | Yes | 701 (87.4) | 12 (1.7) | 689 (98.3) | 1.69 (0.22-13.14) |  |
| Household size | 1-3 | 302 (37.7) | 6 (2.0) | 296 (98.0) | REF | 0.59 |
|  | 4-6 | 366 (45.6) | 5 (1.4) | 361 (98.6) | 0.68 (0.21-2.26) |  |
|  | 7 or more | 129 (16.1) | 1 (0.8) | 128 (99.2) | 0.38 (0.04-3.23) |  |
| Water Source | Municipal water | 784 (97.8) | 12 (1.5) | 772 (98.5) | REF | -- |
|  | Well/Borehole | 11 (1.4) | 0 (0.0) | 11 (100.0) | -- |  |
|  | Other | 4 (0.5) | 0 (0.0) | 4 (100.0) | -- |  |
| Sanitation | Flush toilet | 520 (64.8) | 7 (1.4) | 513 (98.6) | REF | 0.55 |
|  | Pit latrine | 262 (32.7) | 5 (1.9) | 257 (98.1) | 1.42 (0.45-4.54) |  |
|  | Other | 17 (2.1) | 0 (0.0) | 17 (100.0) | -- |  |
| Livestock exposure in 30 days | No | 657 (81.9) | 12 (1.8) | 645 (98.2) | REF | 0.27 |
|  | Yes | 131 (16.3) | 0 (0.0) | 131 (100.0) | -- |  |
|  | Not Sure | 14 (1.8) | 1 (7.1) | 13 (92.9) | 4.13 (0.50-34.19) |  |
| Season of swab collection | Dry | 560 (69.8) | 10 (1.8) | 550 (98.2) | REF | 0.56 |
|  | Rainy | 242 (30.2) | 3 (1.2) | 239 (98.8) | 0.69 (0.19-2.53) |  |

*Pre-existing conditions were defined as any of the following: chronic kidney disease, chronic obstructive pulmonary disease, diabetes mellitus, coronary artery disease/congestive heart failure, hypertension, neurodevelopmental impairment, substance abuse/dependence, and malignancy.

**P-values <0.05 were considered statistically significant
