# Supplementary material for: Admission screening for extended-spectrum cephalosporin-resistant and carbapenem-resistant Enterobacterales colonization at a referral hospital in Botswana: A one-year period-prevalence survey, 2022–2023
Source: PLOS Glob Public Health. 2025 Oct 28;5(10):e0005018. doi: 10.1371/journal.pgph.0005018 (PMC12561989; doi:10.1371/journal.pgph.0005018)
Supplement: Attachment — Submitted filename: Response to Reviewers.docx [file pgph.0005018.s004.docx]

19 July 2025

Muhammad Asaduzzaman, MD MPH MPhil

Academic Editor

PLOS Global Public Health

**PGPH-D-25-01363: Admission screening for extended-spectrum cephalosporin-resistant and carbapenem-resistant Enterobacterales colonization at a referral hospital in Botswana: A one-year period-prevalence survey, 2022-2023**

Dear Dr. Muhammad Asaduzzaman and Reviewers,

We thank you for your constructive and insightful comments on our manuscript. We appreciate the opportunity to revise our submission, and we believe the changes have substantially improved the quality and clarity of the work.

Below, we provide a detailed, point-by-point response to the comments raised by the editor and reviewers. All changes are clearly marked in the revised manuscript with track changes, and we have also submitted a clean version for review.

Journal Requirements

*In the ethics statement in the Methods, you have specified that verbal consent was obtained. Please provide additional details regarding how this consent was documented and witnessed, and state whether this was approved by the IRB.*

Consent was documented and witness by study personnel and this approach was approved by IRB*.* We have now specified this in the Ethics Statement in the revised version of the manuscript.

Reviewer #1 Comments

1. *Lines 118-119: Why were patients excluded if they were in the ER for over 24 hours? Also, why might patients be in the ER for this long? Specifically, are there specific populations (e.g. teenagers) who might be kept in the ER for longer? I am wondering if this exclusion criteria might bias your final study population in some way.*

Distinguishing which patients were already colonized upon arrival to the ED versus those who acquired colonization in the hospital was critical to determining associations between community vs. hospital-associated colonization. Since it is known that acquisition of colonization can happen rapidly in the hospital setting, we excluded those patient which had spent more than 24 hours in the ED so as to avoid differential misclassification.

The reasons for long stays in the ED are multifactorial and related to limited bed space and staff to accommodate the number of admissions. Occasionally, patients are awaiting results from diagnostic work-ups which could dictate whether they would be admitted. In this hospital, since the ED is not staffed with pediatricians or pediatric emergency medicine specialist, pediatric admitting teams often prioritize timely admission to the pediatric wards, thus children are more likely to be sent to patient wards sooner that adult patients. This may have contributed to the higher relative enrolment observed among children and thus may have introduced selection bias. We have updated the limitations section of the manuscript to reflect this possible selection bias.

2. *Lines 130 -133* *Please include citations for these MIC cut-offs to define resistance. The MIC cut-off for ceftazidime in particular seems quite high. Is it possible you missed ESCrE with lower levels of resistance to cefotaxime or ceftazidime?*

Low level resistance is always a possibility but we cited the Clinical and Laboratory Standards Institute (CLSI 100, 34^th^ edition, 2024) as the source for the MIC breakpoints, which is a widely recognized authority on sensitivity cutoffs.

Reviewer #2 Comment

1. *Generalisability:*

*- Single-Centre Limitation: The study is conducted at a single tertiary hospital, which may limit generalizability to other healthcare settings or the broader community in Botswana and sub-Saharan Africa.*

*- Selection Bias: Patients admitted through the Emergency Department may not represent all hospital admissions, especially as direct ward admissions (e.g., maternity) are excluded.*

- We agree that the single-centre design may have limit generalizability. This was mentioned in the limitations section.
- We agree that patients admitted through the Emergency Department may not represent all hospital admissions, particularly the maternity population. This has been the impetus for birth cohort study we are currently carrying at the same hospital focusing specifically on perinatal acquisition of ESCrE/CRE colonization as this was a missed population in the study.

2. *Exposure and Confounding Variables*

*- Incomplete Capture of Healthcare Exposure: The study does not account for non-inpatient healthcare exposures (e.g., outpatient or clinic visits), which may be significant, especially for infants.*

*- Antibiotic Exposure Assessment: Reliance on patient recall for antibiotic use could introduce misclassification bias. Consider discussing potential impact or alternative approaches (e.g., pharmacy records, biomarker testing).*

- We have noted in the limitations, lines 305 – 307, that “there may be unmeasured confounders such as non-inpatient healthcare exposures (e.g., clinic visits) and indirect antibiotic exposure through food and water sources that were not captured.”
- In the Discussion section, we acknowledge that relying on participant recall may have led to misclassification. We note that, “Some studies have used antibiotic residue detected in urine to demonstrate that indirect antibiotic exposures may be a potentially insidious driver of AMR”. However, this was outside of the scope of this study.

3. *Statistical Power for CRE Analysis*

*- Low CRE Prevalence: With only 12 CRE-colonised patients, the power to detect associations is limited, and multivariable analysis was not performed. This limitation is acknowledged but should be emphasised further in the discussion.*

- In the Discussion sections we now write, “Although low CRE colonization resulted in inadequate statistical power to detect associations, the crude association between CRE colonization with and antiretroviral drug exposures warrants further exploration.”

4. Interpretation of Antiretroviral Association
   - Causality and Mechanism: The observed association between antiretroviral drug use and CRE colonisation is intriguing but remains unexplained—the lack of HIV status and viral load data limits interpretation. The discussion should more clearly state that causality cannot be inferred and that this finding is hypothesis-generating.

- Importantly we have included in the Discussion section that, “…because HIV status and viral load were not captured in this study, it remains unclear whether the observed effect is attributable to the use of antiretrovirals (for either therapy or prophylaxis), more frequent outpatient healthcare contact, the underlying immunological effects of HIV, or any combination of the these factors…This finding highlights the need for additional research to disentangle the roles of HIV infection and antiretroviral drugs in driving CRE colonization.”

5. *Minor Concerns and Suggestions*

*- Clarity in Tables: Some tables are dense; consider summarising key findings in the text or using graphical abstracts for clarity.
- Terminology: Ensure consistent use of terms (e.g., "well/borehole" vs. "well water") throughout the manuscript.
- Supplementary Data: Ensure that all supplementary tables and figures are referenced in the main text.
- Formatting: Minor typographical errors (e.g., misplaced line breaks, HTML entities like < and >) should be corrected before publication.*

- All instances of “well water” have been revised to “well/borehole water” for consistency and we have proofread the document to identify any missed typos.

We sincerely appreciate the helpful feedback from the editor and reviewers. We hope the revised manuscript now meets the criteria for publication in PLOS Global Public Health.

Sincerely,

Tshiamo Zankere

Botswana–UPenn Partnership

244G – Room 103

PO Box 45498

Gaborone, Botswana

Email: zankere2016@gmail.com
